# Persistent wheezing as manifestation of esophageal tubular duplication cyst

**DOI:** 10.3109/03009734.2011.574165

**Published:** 2011-06-29

**Authors:** Slobodanka Petrović, Radmila Ljuština, Jovan Lovrenski, Ivan Milović, Nenad Barišić

**Affiliations:** ^1^Department of Pulmology, Institute for Child and Youth Health Care of Vojvodina, Novi Sad, Serbia; ^2^Department of Radiology, Institute for Child and Youth Health Care of Vojvodina, Novi Sad, Serbia; ^3^Institute for Child and Mother Health Care ‘Dr Vukan Čupić’, Belgrade, Serbia; ^4^OINT, Institute for Child and Youth Health Care of Vojvodina, Novi Sad, Serbia

**Keywords:** Anomaly, esophageal cyst, wheezing

## Abstract

Duplications of esophagus are rare congenital anomalies and the second most common duplications of the gastrointestinal tract. This form of bronchopulmonary foregut malformation may appear as a cystic mediastinal mass. On chest radiographs they may be visible as middle or posterior masses. On CT they are well marginated and oppose the esophagus. Usually they are asymptomatic, unless they become infected or cause obstruction. We report a case of thoracic tubular duplication cyst in a 10-month-old boy who presented with persistent wheezing that was unsuccessfully treated in out-patient services.

## Introduction

Embryonal foregut duplication cysts are uncommon lesions that constitute 15% of all mediastinal masses, commonly found in the posterior mediastinum. Some 10%–20% of these anomalies are esophageal duplications (1,2). It can occur all along the esophagus, but a majority is found in a thoracic segment. Esophageal duplication cyst is a rare disorder making up 1 out of 15 cases of mediastinal cysts (3). There are three types of cysts: cystic, tubular, and diverticular. The tubular form belongs to normal esophagus (4,5). Almost all of the reported cases were detected in early days of life or during infancy. Patients with esophageal duplications usually present either with respiratory distress because of airway compression due to an enlarging mass, or with a thoracic mass found incidentally on chest X-ray (CXR) in asymptomatic patients.

The aim of this report is to present an infant with this anomaly and persistent wheezing as a marked clinical sign, unrecognized as a problem in out-patient practice.

## Case report

A 10-month-old male infant was referred to hospital because of prolonged, rough and intensive cough and persistent wheezing refractory to inhaled bronchodilators and corticosteroid therapy. He was unsuccessfully treated 8 weeks before admission. The boy was previously healthy. Because of the broncho-obstruction, as well as positive family history for atopic diseases (mother's asthma), the out-patient pediatrician had already carried out prick test to nutritive allergens (negative), eosinophil count in nasal secretions (negative), and eosinophils in blood (normal). Acute episodes of marked broncho-obstruction occurred 2–3 times per month, with continuously present coughing and mild wheezing in the ‘symptom-free’ periods.

The child was in a good condition. He had no swallowing difficulties or cyanosis. He was normothermic, respiratory rate was 36/min, heart rate was 120/min, SatO_2_ was 96% (by pulse oximetry). Rough and irritable cough was notable, as well as marked persistent wheezing with signs of dyspnea (intercostal and suprasternal retractions).

All additional examinations were normal: C-reactive protein (CRP), total leukocyte count, and differential white blood cell count, along with other biochemical findings (blood glucose levels, serum electrolyte levels, total serum protein, serum immunoglobulin levels, blood gas analysis), ECG and echocardiography findings, chloride in sweat(sweat test), and plain chest X-ray (CXR).

During the first 6 days after admission, the patient was treated with beta-2 agonists and corticosteroids systemically, but wheezing and respiratory distress persisted.

**Figure 1.**
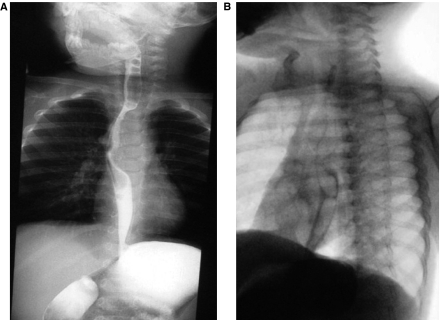
Esophageal fluoroscopy.A:anteroposterior radiograph;B: lateral radiograph.

On the third day of hospitalization esophageal fluoroscopy was performed, showing impression at the middle third of the esophagus, its significant narrowing and shifting to the right. Nevertheless, its wall remained smooth. Diameters of the proximal and distal thirds of esophagus were normal (Figure 1A).

Lateral radiography (Figure 1B) showed an impression at the middle third of the posterior esophageal wall, with its narrowing, but the esophageal contour remained smooth. Also, a narrowing of the distal trachea was noticed. The passage of contrast through the esophagus was normal. Gastroesophageal reflux (GER) was not present.

**Figure 2.**
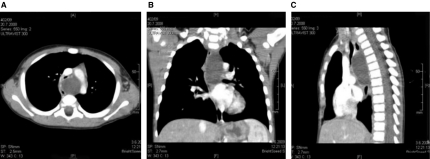
Contrast-enhanced computed tomography (CECT) of the thorax with multiplanar reconstructions. A: axial scan;B: coronal scan;C: sagittal scan.

Following these findings, contrast-enhanced computed tomography (CECT) was performed. It showed a hypodense, well circumscribed, oval-shaped mass with a thin rim located in upper and middle-posterior mediastinum. Its greatest diameter was around 5 cm. The lesion was displacing the esophagus to the right and aorta to the left. The distal third of the trachea was compressed and shifted to the right. There has not been any communication of the mass with the surrounding mediastinal structures seen. In the lungs and the rest of the mediastinum the CT findings were normal. Based on this finding the radiologist raised a suspicion of the tubular form of an esophageal duplication cyst. A bronchogenic cyst was also considered as differential diagnosis (Figure 2).

The decision about surgical treatment was made after consultations including pediatrician, radiologist, and surgeons. Bronchoscopy was performed under general anesthesia, before surgical intervention. Endoscopy revealed marked signs of posterior wall compression in the left principal bronchus with a slit-like bronchial lumen.

The patient underwent a left thoracotomy which revealed a large cystic mass in the posterior mediastinum. Next to the esophagus, a tubular duplication cyst with thin walls was found. Its size and position matched completely the CT and endoscopic findings. The cyst wall was excised in total, except the part which was in direct contact with the esophagus and had a joint wall (unroofing). Integrity of the entire length of the esophagus was checked by palpitation with probe. Biopsy findings were consistent with esophageal tubular duplication cyst.

After the intervention, the respiratory symptoms disappeared. Unfortunately, the patient had a complication of the surgical intervention, and as a result of intraoperative lesion of ductus thoracicus he developed chylothorax.

The child was discharged 6 weeks after surgical treatment, with normal CXR, without symptoms of respiratory or gastrointestinal disease, and normal physical findings.

After 18 months the child is still well and regularly followed up.

## Discussion

Esophageal duplication cysts are benign, asymptomatic bronchopulmonary foregut malformations. Most of them are found by accident, in children evaluated for other medical problems. Estimated incidence of congenital esophageal cyst is 1:8200, with male sex predominance (2:1) (6). Depending on the location and size of the lesion, they may cause compression and obstruction of surrounding structures or infection and bring on presenting symptoms. Gastrointestinal and infectious symptoms are most common. However, if the mass impinges on the trachea, respiratory symptoms may occur. When the cyst becomes symptomatic, its complications may be life-threatening (4,7).

Esophageal duplication can be associated with other congenital anomalies, such as small intestine duplications, esophageal atresias distal to the duplication, tracheoesophageal fistulas, and spinal abnormalities (8).

Our patient did not have any associated anomalies. This infant presented with severe wheezing refractory to all standard modalities of treatment. Usual conditions leading to wheezing were considered as differential diagnosis; the child was fully examined, and a cystic mass that could belong to the esophageal duplication cyst was discovered in the posterior mediastinum. Usually, these cysts are found accidentally on the CXRs in asymptomatic patients (9).

However, when they become symptomatic with different features, mostly respiratory, it is often a sign of development of complications (infections, displacement and/or compression of adjacent organs, hemorrhage, rupture, or neoplasia) (10). In our patient, signs of respiratory distress and persistent wheezing were caused by compression of airways. Different diseases are presented with broncho-obstructive symptoms, and usually these children are considered to have viral wheezing (in infancy) or atopic asthma. Prolonged treatment in out-patient services can be a problem, allowing development of serious complications.

Non-invasive radiological methods can be helpful in the diagnosis. In these cases barium esophagogram is useful, revealing compression of the esophagus. CT has the advantage over conventional diagnostic procedures because it demonstrates the exact anatomic position of the cyst, nature of the mass, its relationship to the adjacent structures, and also influences the decision-making about resectability (11,12).

A symptomatic cyst is an indication to immediate resection. Surgical excision is recommended at the time of discovery to prevent the development of complications and to avoid the potential malignant transformation (2).

## Conclusion

Duplication cysts are rare but should be included in the differential diagnosis of persistent wheezing. A high index of suspicion is key to early diagnosis and treatment, because cyst progression in children can rapidly lead to obstruction and severe respiratory failure.
